# Sperm Mitochondrial Content and Mitochondrial DNA to Nuclear DNA Ratio Are Associated with Body Mass Index and Progressive Motility

**DOI:** 10.3390/biomedicines11113014

**Published:** 2023-11-09

**Authors:** Efthalia Moustakli, Athanasios Zikopoulos, Charikleia Skentou, Ioanna Bouba, Georgia Tsirka, Sofoklis Stavros, Dionysios Vrachnis, Nikolaos Vrachnis, Anastasios Potiris, Ioannis Georgiou, Athanasios Zachariou

**Affiliations:** 1 Laboratory of Medical Genetics, Faculty of Medicine, School of Health Sciences, University of Ioannina, 45110 Ioannina, Greece; ioannabouba@gmail.com (I.B.); ge.tsirka@gmail.com (G.T.); igeorgio@uoi.gr (I.G.); 2 Obstetrics and Gynecology, Royal Cornwall Hospital, Truro TR1 3LJ, UK; thanzik92@gmail.com; 3 Department of Obstetrics and Gynecology, Medical School of Ioannina, University General Hospital, 45110 Ioannina, Greece; haraskentou@gmail.com; 4 Third Department of Obstetrics and Gynecology, Attikon Hospital, Medical School, National and Kapodistrian University of Athens, 12462 Athens, Greece; sfstavrou@med.uoa.gr (S.S.); nvrachnis@hotmail.com (N.V.); apotiris@med.uoa.gr (A.P.); 5 Department of Clinical Therapeutics, Alexandra Hospital, Medical School, National and Kapodistrian University of Athens, 12462 Athens, Greece; dionisisvrachnis@gmail.com; 6 Vascular Biology, Molecular, and Clinical Sciences Research Institute, St George’s University of London, London SW17 0RE, UK; 7 Department of Urology, School of Medicine, Ioannina University, 45110 Ioannina, Greece; zahariou@otenet.gr

**Keywords:** sperm quality, obesity, male infertility, reproductive health, oxidation, IVFI/ICSI, ART

## Abstract

Background: Mitochondrial dysfunction is a risk factor in the pathogenesis of metabolic disorders. According to the energy requirements, oxidative phosphorylation and the electron transport chain work together to produce ATP in sufficient quantities in the mitochondria of eukaryotic cells. Abnormal mitochondrial activity causes fat accumulation and insulin resistance as cells require a balance between the production of ATP by oxidative phosphorylation (OXPHOS) in the mitochondria and the dissipation of the proton gradient to reduce damage from reactive oxygen species (ROS). This study aims to explore the relationship between the mitochondrial content of sperm and the ratio of mitochondrial DNA to nuclear DNA in relation to body mass index (BMI) and how it may affect the progressive motility of sperm cell. Understanding the relationships between these important variables will help us better understand the possible mechanisms that could connect sperm motility and quality to BMI, as well as further our understanding of male fertility and reproductive health. Methods: Data were collected from 100 men who underwent IVF/ICSI at the University Hospital of Ioannina’s IVF Unit in the Obstetrics and Gynecology Department. The body mass index (BMI) of the males tested was used to classify them as normal weight; overweight; and obese. Evaluations included sperm morphology; sperm count; sperm motility; and participant history. Results: In the group of men with normal BMI, both BMI and progressive motility displayed a statistically significant association (*p* < 0.05) with mitochondrial DNA content, relative mitochondrial DNA copy number, and the mtDNA/nDNA ratio. Similar to this, there was a positive association between BMI and motility in the groups of men who were overweight and obese, as well as between the expression of mitochondrial DNA and the mtDNA/nDNA ratio, with statistically significant differences (*p* < 0.05). There was not a statistically significant difference observed in the association between the relative mtDNA copy number and BMI or motility for the overweight group. Finally, the relative mtDNA copy number in the obese group was only associated with motility (*p* = 0.034) and not with BMI (*p* = 0.24). Conclusions: We found that in all three groups, BMI and progressive motility exhibited comparable relationships with mitochondrial DNA expression and the mtDNA/nDNA ratio. However, only in the normal group and in the obese group, the relative mitochondrial DNA copy number showed a positive association with BMI and progressive motility.

## 1. Introduction

In the midpiece of the human sperm, there are between 50 and 75 mitochondrial fragments organized end to end, helically encircling the outer dense fibers of the tail [1]. The sperm mitochondria are rendered mechanically stable by the mitochondrial capsule, which also makes mitochondria resistant to the hypoosmotic environment [2]. Sperm mitochondria are essential for spermatozoa’s functionality because they produce cell energy for sperm motility and control cell death [3].

The number of mitochondrial DNA (mtDNA) copies per nuclear DNA copy is known as the sperm mitochondrial DNA copy number (mtDNAcn), and it is a significant mitochondrial genetic characteristic [4]. The absence of protective histones and the ability to repair DNA in mtDNA, as opposed to nuclear DNA, makes mtDNA more susceptible to harmful influences [5]. Interestingly, paternal mtDNA composes only a minor proportion of the mtDNA within the fertilized oocyte, although the father’s mitochondria enter the egg during fertilization. Therefore, any changes to the mtDNA could have an impact on how fertilization normally occurs [6].

Although they are relatively uncommon, mitochondrial-associated problems that can impair male fertility can have a serious effect on male reproductive health [7]. Sperm motility issues, low sperm counts, oxidative stress, asthenozoospermia, genetic mitochondrial abnormalities, and age-related decline are a few mitochondrial-related problems that may lead to male infertility [8]. The likelihood of mitochondrial problems in sperm increases with increasing paternal age. Although mitochondrial problems can contribute to male infertility, it is vital to remember that they are frequently only one of a variety of factors affecting men’s fertility [3,8].

Sperm mitochondrial dysfunction can be caused by a variety of clinical disorders as well as lifestyle factors. This dysfunction can affect sperm motility, viability, and DNA integrity by reducing ATP synthesis and increasing ROS levels [9]. Increased oxidative stress caused by mitochondrial dysfunction can damage sperm cell membranes, proteins, and DNA. This can result in fertility problems and reduced sperm quality. Mitochondrial dysfunction can result from mtDNA mutations [10].

Sperm quality is greatly influenced by mitochondrial function, and abnormalities in this function can have a substantial impact in both pathological and physiological conditions. Two physiological conditions in which mitochondria function are energy production and ROS control [11]. This energy is produced by the mitochondria in the middle part of the sperm tail as adenosine triphosphate (ATP). The energy required for sperm motility must be provided by functional mitochondria. Both the acrosome reaction and capacitation, which are necessary steps for fertilization, normally require a regulated amount of ROS. In order to maintain the delicate balance between ROS levels that promote sperm activity and oxidative stress, which can damage sperm DNA, mitochondria participate in the regulation of ROS production [3].

Research indicates that both underweight and overweight or obese people can be impacted by changes in reproductive health, including in the quality of sperm. Males with a healthy BMI tend to have higher sperm counts and motility than underweight males [12]. The production and quality of sperm can be negatively affected by hormonal imbalances linked to obesity, such as elevated estrogen and decreased testosterone levels. Insulin resistance can also result from obesity. Obesity is frequently linked to increased oxidative stress and chronic inflammation in the body [13].

Elevated mtDNA mutation rates, cell damage, and mortality are all possible outcomes of increased reactive oxygen species (ROS) generation. The proton gradient cannot be efficiently dissipated due to several parameters, including excessive calorie intake, an increase in the substrate load on the mitochondria, or a malfunction of the mitochondria [14]. To gain further insight into how metabolic and genetic factors may affect male reproductive health, this study aims to investigate the relationships between sperm mitochondrial content, the mitochondrial DNA to nuclear DNA ratio, and their associations with body mass index (BMI) and sperm progressive motility.

## 2. Materials and Methods

### 2.1. Participants’ Characteristics and Semen Sample Collection

Our study included 100 men who underwent IVF/ICSI in total. The IVF Unit of the Obstetrics and Gynecology Department of the University Hospital of Ioannina, Ioannina, Greece, provided the semen samples. Thirty of the men had normal body mass index (ranging from 18 to 24.9 kg/m^2^), thirty-eight men were overweight (ranging from 25 to 29.9 kg/m^2^), and thirty-two men were obese (class I and II) with a body mass index higher than 30 kg/m^2^. According to their body mass indices, which were classified as normal, overweight, and obese, respectively, participants were later stratified into three groups. Each participant signed the informed consent form. The characteristics of each sperm sample were identified using Kruger’s criteria. The men in the control group had normal BMIs and sperm parameters that met Kruger’s standards for normality. As well as these details, each participant’s sperm count, morphology, motility, DNA fragmentation index, and conditions such as varicocele, drug usage, and smoking were also noted.

According to Kruger et al., tougher sperm morphology standards may be a reliable indicator of infertility during in vitro fertilization (IVF). According to Kruger’s requirements, the sperm head must be oval-shaped, regular, and have a smooth, constant outline. Additionally, the acrosome must be intact. Furthermore, midpiece and tail abnormalities are not permitted. Another indication of morphological issues may be bent, numerous, or short tails as well as coiled, bowed, or sloppy midpieces. Finally, a high cytoplasmic droplet count may be a sign of sperm immaturity [15].

### 2.2. Sperm DNA Extraction and Quantification

First, a fresh semen sample was required for the measurement of sperm parameters such as sperm count, motility, morphology, and DFI. Within an hour of ejaculation, the samples were examined using the Computer Aided Semen Analysis (CASA) system to accurately gauge motility. For the analysis, we used the Zeiss^®^ Lab.A1 electron microscope, a BASLER^®^ camera, Jena, Germany, and the CASA SPERM CLASS ANALYZER (SCA) SCA 6 Evolution software from Microptic Automatic Diagnostic Systems SL (Barcelona, Spain). The samples were then frozen at a temperature of −20 °C for storage. Since freezing does not affect the quality of DNA, samples do not need to be fresh to extract DNA. The Qiagen methodology was used in the current investigation along with a matching kit; the QIAmp DNA blood Mini kit. During the first stage, 100 μL of semen sample was combined with an X2 buffer and heated for at least an hour at 56 °C in the heat block. Each sample was vortexed at regular intervals. Following this, a volume of 200 μL from each preheated semen sample was processed for sperm DNA extraction using the QIAmp DNA blood Mini kit according to the manufacturer’s instructions.

The extracted sperm DNA was eluted in 80 μL of elution buffer and quantified by NanoDrop Spectrophotometer (Thermo Fisher Scientific, Waltham, MA, USA) to measure purity and concentrations. The purity of the extracted DNA was assessed by measuring the A260/A80 ratio via spectroscopy for DNA analysis, both nuclear and mitochondrial-specific primers were used. The human retinoid isomerohydrolase gene (RPE65) served as the nuclear DNA reference gene for the quantification of nDNA using the RPE primers 5′-ATAGGAAGCCAGAGAAGAGAGACT-3′ and 5′-TCTATCTCTGCGGACTTTGAGCAT-3′, (200 bp). Meanwhile, the combination of primers 5′-TAGAGGAGCCTGTTCTGTAATCG-3′ and 5′-TAAGGGCTATCGTAGTTTTCTGG-3′, (205 bp) was utilized for the mitochondrial DNA quantification, which corresponds to a section of the mitochondrially produced 12 S RNA (Table 1).

### 2.3. Real-Time Quantitive PCR–qPCR

The quantitative polymerase chain reaction (qPCR) was used to assess the mtDNA/nDNA ratio. For each reaction, we combined 0.02–0.03 μg of extracted DNA, 0.25 μL of each 10 μM forward and reverse primer, 5 μL of SYBR Green Master Mix (PowerUp SYBR Green Master Mix, Applied Biosystems, Thermo Fisher Scientific, USA), 2.5 μL of distilled water, and a total volume of 10 μL. The reaction was performed on a Corbett Rotor-Gene 3000 Real-Time Rotary Analyzer (Corbett Research, Sydney, Australia) under the following cycling conditions: 50 °C for 2 min, 95 °C for 2 min, followed by 40 cycles of denaturing at 95 °C for 15 s and 60 °C for 1 min. Each sample was examined in duplicate. The relative mitochondrial DNA content per sperm sample was then determined by using the ΔΔCt technique, which is explained below, to compute the ratio of mtDNA to nDNA.

We chose a control group and applied the cycle threshold Ct values as follows for the relative quantification (also known as the “ΔΔCt method”) [16,17]:

(a) By deducting the mean mitochondrial Ct value from the mean nuclear Ct value for each sample as indicated ΔCt = mtDNA − nDNA;
(b) The average ΔCt value for the control group, which in this instance consisted of men with normal body mass index (18.5 to 24.9 kg/m^2^) and normal semen characteristics;
(c) The ΔΔCt for each sample by subtracting the ΔCt of the control group from the sample’s mean ΔCt, that is, ΔΔCt = ΔCt of a particular sample minus the ΔCt of the control group [17];
(d) By applying the formula 2^−ΔΔCt^, the fold difference.

The ΔΔCt values for each sample are calculated by deducting the ΔCt of the control group from the sample’s mean ΔCt, or ΔΔCt = ΔCt of a particular sample minus the Ct of the control group. Men with normal body mass index (18.5 to 24.9 kg/m^2^) and normal semen parameters were used as the control group. The equivalent values (mean SD) were as follows: for men with normal BMI and abnormal and normal semen characteristics; for men who were overweight and had normal and abnormal semen characteristics; and for men who were obese.

### 2.4. Statistical Analysis

**Scheme 1:**
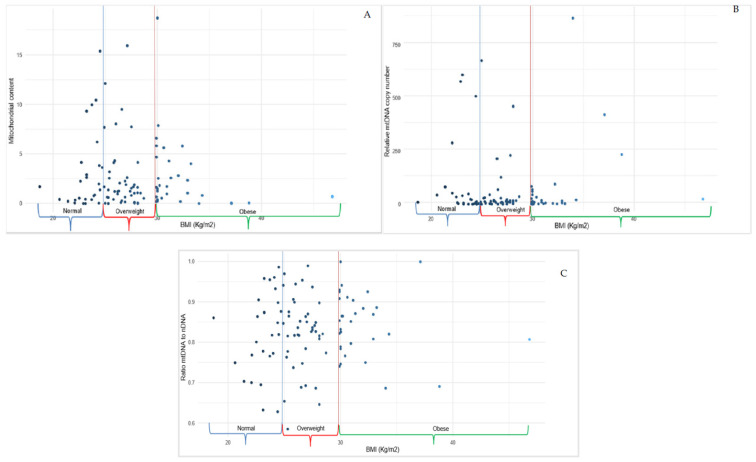
The graphs above display the overall change in the three parameters analyzed in terms of body mass index. The diagrams are arranged in the following order: (**A**) the mitochondrial content; (**B**) the relative mitochondrial DNA copy number; and (**C**) the ratio of mitochondrial to nuclear DNA. The statistical software R was used to create the plots.

To determine the *p*-values for the data comparing three groups, we performed ANOVA analysis. The Spearman’s correlation coefficient was used to create the scatter plots. Microsoft Excel 2016 spreadsheets were used to organize the extraction data, mtDNA copy numbers, and Ct rates to create graphs. The R statistical computing program was used to create the plots shown in the Scheme 1. Statistical significance was defined as *p* < 0.05.

### 2.5. Inclusion and Exclusion Criteria

The table below (Table 2) displays the study’s inclusion and exclusion criteria. 

## 3. Results

Three groups of participants were stratified based on body mass index. The Table 3 below displays the age, the number of participants tested in each group, as well as certain sperm parameters like motility, sperm count, and morphology.

The following graphs depict the distribution a. of mitochondrial content, b. the relative mitochondrial DNA copy number, as well as c. the ratio of mitochondrial to nuclear DNA for the three groups under study, normal, overweight, and obese.

The average mitochondrial content, the relative mtDNA copy number, and progressive motility in each group under study (normal, overweight, and obese) are represented graphically. Compared to the other groups, the normal group exhibited higher levels of mtDNA content and progressive motility. According to the relative mtDNA copy number in comparison to the three categories, the percentage of the overweight group was almost twice as high as that of the normal group and three times higher than that of the obese group. Additionally, we notice that the error bars also display negative numbers. There are no negative values for the relative copy number of mitochondria. The fact that the error bar for overweight is negative suggests that our data are somewhat variable and that the genuine population mean may be lower than the sample mean.

### 3.1. Assessment of Mitochondrial DNA Content

In recent years there has been an increase in scientific reports on the association between sperm mitochondrial content and body mass index (BMI). Mitochondria are vital organelles in cells that are inherited from the mother through the egg and are critical for the generation of energy. Sperm function and fertility may be impacted by changes in the mitochondrial composition of sperm [18].

The metabolic and hormonal alterations brought on by obesity are thought to be connected to the impact of BMI on sperm mitochondrial content. Obesity can cause oxidative stress and systemic inflammation, both of which can have an impact on the mitochondrial processes in many tissues, including sperm. Individual differences may exist in the correlation between BMI and sperm mitochondrial load. Obesity does not always result in altered sperm mitochondrial composition or fertility [19].

**Figure 1:**
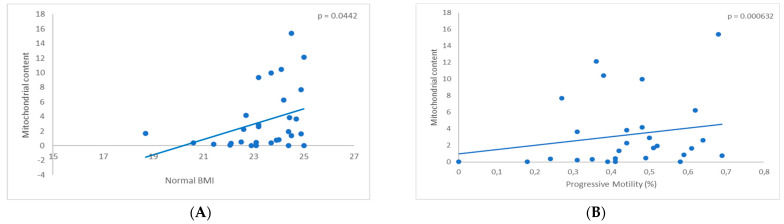
Evaluation of mitochondrial DNA content with BMI (**A**) and progressive motility (**Β**) in the group of individuals with normal BMI. Statistically significant results were found for both parameters (*p* = 0.0442 and *p* = 0.000632, respectively).

**Figure 2:**
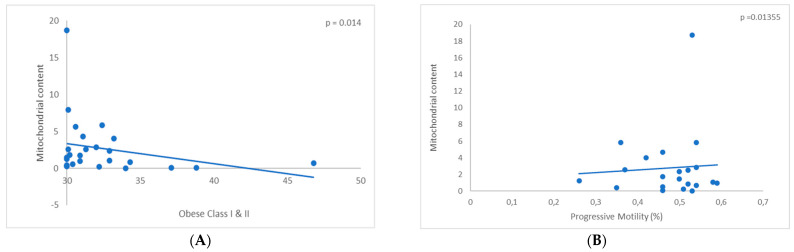
A comparison of the obese group’s mitochondrial load, BMI ((**A**), *p* = 0.014), and motility ((**Β**), *p* = 0.01355). These parameters are each statistically significant, *p*-values < 0.005.

**Figure 3:**
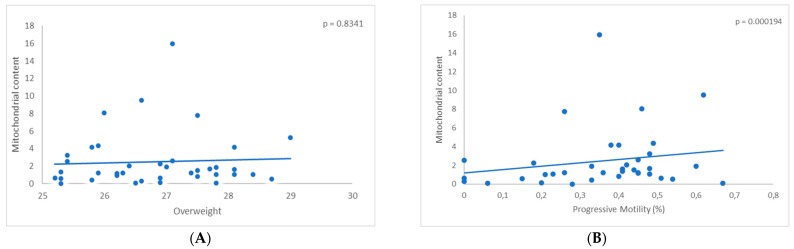
Assessment of mitochondrial DNA content in the overweight group about BMI ((**A**), *p* = 0.8341) and motility A + B ((**Β**), *p* = 0.000194).

The findings relating to the mitochondrial DNA content of the BMI groups and the motility in the three examined groups are shown below. The mitochondrial content rises in the three study groups as the BMI rises. The results are statistically significant, with the corresponding *p* values for normal (*p* = 0.0442, Figure 1A) and obese groups (*p* = 0.014, Figure 2A), whereas for the overweight (*p* = 0.8341, Figure 3A) the results were not statistically significant.

Additionally, we observed that sperm motility and mitochondrial concentration are linked. The data we found were roughly the same but similarly statistically significant across the normal and overweight groups, *p*= 0.000632 (Figure 1B) and *p*= 0.000194 (Figure 3B), respectively. The mitochondrial content is primarily dispersed in progressive motility values from thirty to sixty percent in the obese group (*p*= 0.01355, Figure 2B), as can be shown.

### 3.2. Assessment of the Relative mtDNA Copy Number

A useful indicator for determining the number of mitochondria and how well they are functioning within a cell or tissue sample is the relative mtDNA copy number. It can shed light on cellular health, energy metabolism, and how different variables affect mitochondrial biology. To examine a wide range of biological processes and disorders, researchers employ this measurement in both basic scientific research and clinical diagnosis.

**Figure 4:**
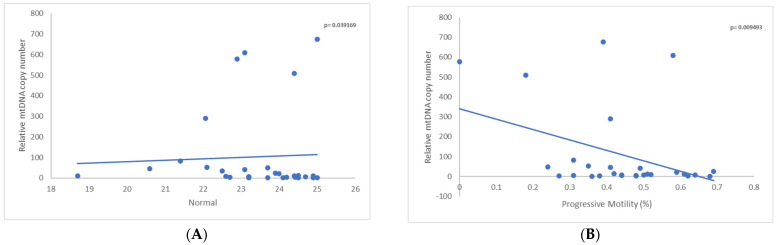
In the normal group, higher normal BMI was associated with a higher relative mtDNA copy number ((**A**), *p* = 0.039169), whereas higher motility copies ranged from 0.3 to 0.7 ((**Β**), *p* = 0.009493).

**Figure 5:**
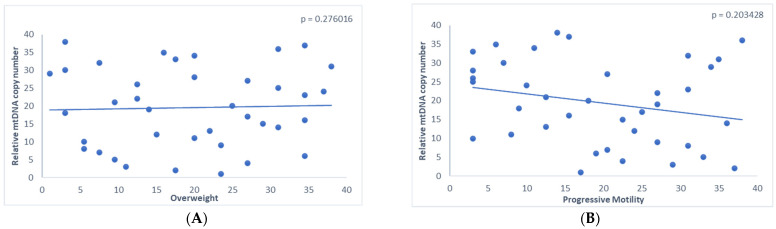
In the overweight group, there was a positive correlation between the relative mtDNA copy number and BMI (**A**) while there was a negative association when compared with progressive motility (**B**). Both showed no statistically significant association (**A**) *p* = 0.276016 and (**B**) *p* = 0.203428.

**Figure 6:**
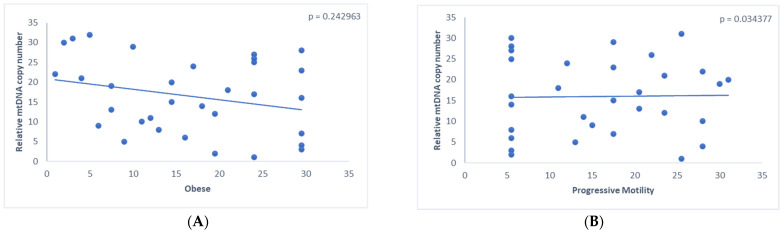
In the obese group, the comparison between the relative mtDNA copy number and BMI illustrated a negative correlation with no significant difference ((**A**), *p* = 0.242963). The relationship between relative mtDNA copy number and progressive motility was positively associated but with no statistically significant difference ((**B**), *p*= 0.034377).

Only in the group with normal body mass index did a link between relative mitochondrial copy number and ΒΜΙ emerge, *p* = 0.039 (Figure 4A). However, it appears that the relative mtDNA copy number did not correlate with BMI in the groups of men who were overweight (*p* = 0.27, Figure 5A) and obese (*p* = 0.24, Figure 6A).

The correlation between the relative mtDNA copy number and progressive motility was next assessed. With *p* values of 0.0094 (Figure 4B) and 0.034 (Figure 6B), respectively, it appears that there is a correlation between these two parameters in both the normal group and the obese group. On the other hand, the relative mtDNA copy number in the overweight group is not correlated with motility A + B (*p* = 0.20, Figure 5B).

### 3.3. Assessment of the Ratio mtDNA to Nuclear DNA

The ratio of mitochondrial DNA (mtDNA) to nuclear DNA (nDNA) can reveal crucial information about a variety of biological processes and conditions. This ratio is frequently used as a marker to determine the amount of mitochondria or how well they are functioning within a cell or tissue [17]. The ratio of mtDNA to nDNA can reveal information on the presence and function of mitochondria, cellular stress and damage, disease states, and responses to environmental factors [5].

**Figure 7:**
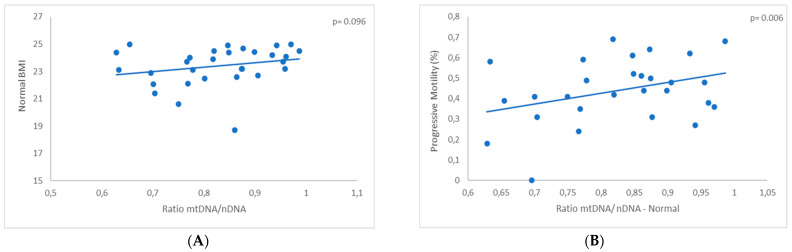
For the group with a normal BMI, the mtDNA/nDNA ratio for both parameters, BMI (**A**) and motility A + Β (**Β**), varied between 0.65 and 1 (*p* = 0.096 and *p* = 0.006, respectively).

**Figure 8:**
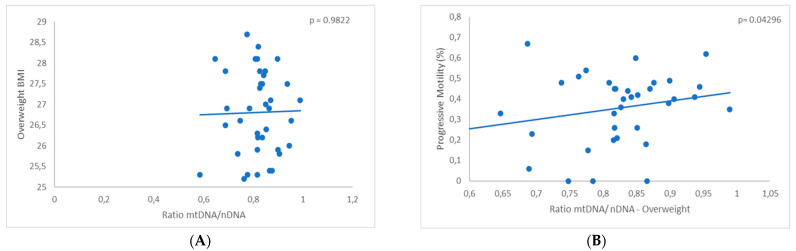
In terms of BMI, the ratio in the overweight group exhibited a more consistent range around the value of 0.8 (**A**), whereas for motility A + B, we noted variances between 0.6 and 1 (**Β**). Statistically significant results were found for progressive motility and BMI (*p* = 0.9822 and *p* = 0.04296, respectively).

**Figure 9:**
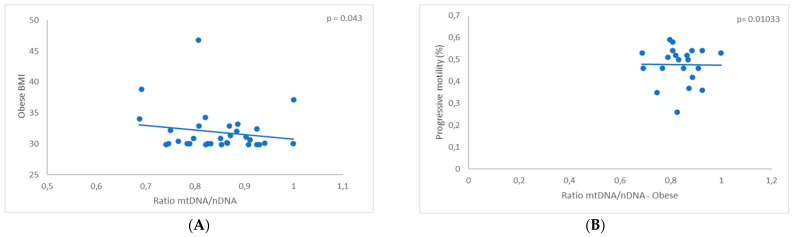
The mtDNA/nDNA ratio for both BMI ((**A**), *p* = 0.043) and progressive motility ((**Β**), *p* = 0.01033) showed a variation of values between 0.7–1. The outcomes were significant for both parameters equally.

When comparing BMI with the mtDNA/nDNA ratio, we found outcomes of statistically significant significance in normal and obese groups. The BMI ranges in the normal group between 0.6 and 1 mtDNA/nDNA ratio (*p* = 0.096, Figure 7A). The BMI ratio appears to be distributed around 0.8 in the overweight population graph (*p* = 0.9822, Figure 8A), whereas the ratio is distributed between 0.7 and 0.9 in the obese population graph (*p* = 0.043, Figure 9A), respectively.

Finally, the relationship between motility and the mtDNA/nDNA ratio was examined. The distribution of data between the normal and overweight groups was similar, and both groups were statistically significant (*p* = 0.006 (Figure 7B) and *p* = 0.04296 (Figure 8B), respectively). Progressive motility in the obese group exhibits a ratio distribution with statistical significance between 0.8 and 1 values (*p*= 0.01033, Figure 9B).

Data were derived from qPCR analysis of DNA isolated from sperm samples acquired from men attending the hospital for IVF/ICSI treatments and analyzed with the 2^−ΔΔCt^ method. The outcomes revealed that these characteristics correlated favorably.

Progressive motility was associated with the relative mtDNA copy number and mtDNA expression. According to the investigation, motility and mtDNA expression are positively correlated. Further research revealed a negative association between motility and the relative mtDNA copy number.

As indicated by the graphical representation below, there was also a significant difference (*p* < 0.05) between the relative mtDNA copy number in relation to normal BMI and the link between mtDNA/nDNA and progressive motility.

## 4. Discussion

Three variables—mitochondrial content, the relative number of copies of mitochondrial DNA, and progressive motility—were examined in the current study based on the body mass index of the individuals. Body mass index and progressive motility, as well as mitochondrial content and the mtDNA to nDNA ratio, all exhibit positive correlations in the overweight and obese group. The relative copy number, however, was linked with mobility rather than body mass index in the obese group. Overweight and obesity have become major public health concerns worldwide as a result of changes in modern lifestyles, with alarming increases in rates of such individuals in industrialized countries [19]. Globally, 13% of adults are obese and 39% of the population is overweight, according to the latest figures [20]. The environment and genetics both have a role in the metabolic disorder known as obesity, which affects the entire body [21,22].

Furthermore, in both the normal and overweight groups, there was a positive association between progressive motility mitochondrial content and BMI. However, mitochondrial content in the obese group correlated negatively with BMI and positively with progressive motility. In the normal group, there was a positive correlation between motility and BMI as well as relative mtDNA copy number. In the overweight group, there was a positive connection with BMI but a negative correlation with motility. The relative mtDNA copy number, however, correlated positively with motility and negatively with BMI in the obese group [23].

The average human spermatozoon contains one copy of mtDNA per mitochondrion [24]. Although the sperm’s mtDNA sequence is the same as that of somatic cells, the sperm’s DNA-repairing activity is either significantly reduced or nonexistent [25]. It has been demonstrated that, on this account, sperm rapidly accrue mtDNA mutations despite being produced from mitotic cells (spermatogonia) and having a significantly shorter lifespan than somatic cells. [26]. Based on current studies, 84–86% of ‘normal’ viable sperm have defective mtDNA, and the ratio of abnormal to wild-type mtDNA does not correspond to the motility of the sperm [25].

The spread of sperm mtDNAcn has been linked to defective spermatogenesis [27] and a dysfunctional autophagy pathway in the breakdown of mitochondria [28]. Greater sperm mtDNAcn was linked to worse sperm motility, concentration, and total count, according to two cross-sectional investigations among male partners of couples undergoing fertility testing [29,30]. Furthermore, three observational studies showed that spermatozoa from individuals with defective semen parameters significantly amplified mtDNAcn [27,31,32]. These investigations suggest that the sensitive indicator of the integrity of human sperm may be sperm mtDNAcn. Sperm motility may be affected by differences in mtDNA integrity or mitochondrial activity, according to studies [29].

Progressive motility and sperm count are two important parameters used to assess the quality of sperm in a semen analysis [33]. There may be some correlations or reasons for such observations in some circumstances, even if it is typically not true that obese people consistently score higher on these measures [34]. Understanding that individual variance might be significant and that not all obese people will produce sperm of excellent quality is crucial. According to various research or circumstances, the obese group’s high sperm count and progressive motility may be explained by the following factors: hormonal factors, increased testosterone, lifestyle factors, selection bias, study limitations, and genetics [35].

For instance, a computer-assisted sperm analysis system (CASA) was used to evaluate the sperm of 1285 men. In comparison to males of normal weight, obese men showed decreased sperm concentration and motility as well as a greater incidence of sperm head abnormalities and deformities [12]. In addition, obese men typically have a longer time to pregnancy and a lengthier gestation period [36]. These studies revealed a significant link between a rise in male body mass index and a decline in sperm quality in light of this.

Sperm quality, an important variable in its prediction, considerably affects male fertility [37]. Mitochondria are essential for sperm function as they provide the energy required for sperm motility, an important component of fertility [2,38]. Understanding the potential relationships between sperm mitochondrial content and the mtDNA/nDNA ratio body mass index and progressive motility is of great interest. The increasing motility of sperm, which is necessary for the sperm to pass through the female reproductive system to reach and fertilize the egg, depends on an appropriate supply of functional mitochondria [3].

Numerous research studies have examined the connection between sperm quality and BMI. Obesity is known to have a detrimental influence on male fertility, even though the precise effects of BMI on mitochondrial content and the mtDNA/nDNA ratio have not been well-documented [39]. Kozopas and colleagues’ research indicated that as BMI increased, the quality of sperm also declined [40]. However, further research is necessary to determine the precise processes relating the mtDNA/nDNA ratio and mitochondrial content to BMI.

Obesity and a high BMI are two issues that can affect the apoptotic process. Chronic low-grade inflammation is frequently linked to obesity. Adipocytes, or fat cells, are among the cell types in the body that may be impacted by this inflammation. Obese people’s adipocytes have the ability to release adipokines and inflammatory mediators that disrupt regular cell functions and encourage inflammation [41]. Evidence exists to imply that an imbalance between the processes of adipocyte death and adipogenesis may result from obesity. The size and functionality of adipose tissue can be impacted by this equilibrium, which can also lead to metabolic problems associated with obesity [42].

Metabolic dysregulation, encompassing insulin resistance and dyslipidemia, is linked to obesity. These metabolic imbalances can have an effect on different tissues and cells, which may have an effect on apoptosis and cell survival [43]. Cellular alterations and dysregulation are frequently present in these disorders, which might impact cell death and viability in impacted tissues and organs [44].

Moreover, there is data that suggests a connection between a man’s BMI and the quality of his sperm. Obesity, which frequently corresponds to a high BMI, has been shown in several studies to lower sperm counts. The chance of successful fertilization can be lowered by a reduced sperm count [13]. According to certain studies, men who are overweight or obese may have less motility in their sperm when compared to men who have a normal BMI. Abnormal sperm morphology has been correlated with a higher frequency of high BMI, which may compromise the sperm’s ability to penetrate the egg [45,46].

Hormonal imbalances caused by obesity can include low testosterone and high estrogen levels. Sperm quality and production might decrease as a result of these hormone abnormalities. Obesity and oxidative stress are frequently linked to DNA fragmentation and sperm cell damage [13]. One possible cause of decreased sperm quality is oxidative stress. Poor circulation of heat caused by excess body fat can lead to higher scrotal temperatures. High testicular temperatures can impair the quality and quantity of sperm produced. Couples may find it more difficult to conceive if a man has poor quality sperm. Positively, research has indicated that men who are overweight or obese may see an improvement in the quality of their sperm by following a diet and exercising [47].

Male fertility is influenced by sperm mitochondrial concentration and the mtDNA/nDNA ratio, particularly in the context of sperm motility [48]. A new area of research on the connections between these variables, BMI, and progressive motility has the potential to significantly impact our understanding of male reproductive health and the development of therapies to address fertility problems brought on by obesity [29,37]. There is a general understanding of the role of mitochondria in sperm function and the detrimental effects of obesity on male fertility, but more research is still required to establish a precise connection between BMI, mitochondrial content, mtDNA/nDNA ratio, and progressive motility [3].

## 5. Conclusions

As a result, a fascinating element of male reproductive health has been illuminated by research into the associations between sperm mitochondrial concentration, mitochondrial DNA to nuclear DNA ratio, and progressive motility. The results of this study highlight the complex interactions between sperm quality, BMI, and mitochondrial function. Changes in BMI and progressive motility were shown to be associated with higher mitochondrial content and altered mitochondrial DNA to nuclear DNA ratios, indicating that these characteristics may be important for male fertility.

These findings highlight how crucial it is to maintain a healthy BMI for both general health and reproductive health. Furthermore, the connection between mitochondrial health and sperm quality underscores the need for additional studies into potential therapies that could boost male fertility by focusing on mitochondrial health. Overall, this study advances our knowledge of the complex variables affecting the health of male reproduction and lays the groundwork for further investigation into this vital area.

## 6. Limitations of the Study

This study has several major limitations that should be taken into account. The sample size was relatively small, which could have limited the findings’ applicability to a larger population. Furthermore, the data were gathered through self-report surveys, which might result in response bias and mistakes. It is also difficult to establish causal connections or monitor changes over time because the study’s cross-sectional design only offers a snapshot in time. The study also concentrated on a particular group of people or area, which may have limited the results’ application in other situations. Finally, potential confounding factors that might have influenced the associations reported in the study were not thoroughly investigated. When analyzing and using the study’s findings, it is important to keep these limitations in mind.

## Figures and Tables

**Table 1:** The size of the target region, the matching Tm, and the oligonucleotide sequences for cf-Mt/cf-N determination utilized for real-time quantitative PCR.

Primer Pair	Sequence (Forward/Reverse)	Amplicon Base Pair (bp)	Tm
Nuclear region (gene RPE)	Forward 5′-ATAGGAAGCCAGAGAAGAGAGACT-3′ Reverse 5′-TCTATCTCTGCGGACTTTGAGCAT-3′	200	60 °C
Mitochondrial region	Forward 5′-TAGAGGAGCCTGTTCTGTAATCG-3′ Reverse 5′-TAAGGGCTATCGTAGTTTTCTGG-3′	205	59 °C

**Table 2:** Study’s inclusion and exclusion criteria.

Inclusion Criteria	Exclusion Criteria
Males of reproductive age	Males without informed consent
IVF/ICSI participants	Urological conditions (e.g., varicocele)
Lifestyle (smoking, alcohol, drugs etc.)	Abnormal Karyotype
	AZF deletions

**Table 3:** Semen characteristics of the men who participated in the study, including the male age, progressive motility, morphology, and sperm count, CI 95% included. Statistical significance was defined as *p* < 0.05.

Semen Characteristics	Normal	Overweight	Obese	*p*-Values
No of participants (total = 100)	30	38	32	
Male age, CI 95%	32.5 ± 4.93	36.73 ± 5.614	30 ± 3.099	2.39 × 10^−24^
Progressive motility (%), CI 95%	43.93 ± 6.1	40.33 ± 5.0	43.00 ± 3.6	5.26 × 10^−12^
Sperm count, million/mL, CI 95%	36.68 ± 1.421	25.04 ± 1.1137	89 ± 2.2742	0.0068
Sperm morphology (%), CI 95%	13.38 ± 4.6	10.14 ± 6.8	10.00 ± 5.0	2.32 × 10^−10^

## Data Availability

Data is unavailable due to privacy or ethical restrictions.
